# Examining the correlation between sexual and reproductive health stigmatization level and gender perception: a case of a university in Turkey - a descriptive cross-sectional study

**DOI:** 10.1590/1516-3180.2022.0278.03062022

**Published:** 2022-09-12

**Authors:** Filiz Polat, Derya Kaya Şenol

**Affiliations:** I PhD. Assistant Professor, Department of Midwifery, Faculty of Health Sciences, Osmaniye Korkut Ata University, Osmaniye, Turkey.; II PhD. Assistant Professor, Department of Midwifery, Faculty of Health Sciences, Osmaniye Korkut Ata University, Osmaniye, Turkey.

**Keywords:** Sexual health, Reproductive health, Women, Sexual behavior, Gender role, Social marginalizations, Traditional societies, Societal norm

## Abstract

**BACKGROUND:**

Stigmatization, which emerges depending on the sexual behavior of young individuals, leads to negative health and social outcomes, such as shame, social marginalization, violence, and mental health morbidity.

**Objective:**

This study aimed to examine the correlation between the level of sexual and reproductive health stigma and gender perception in female university students.

**DESIGN AND SETTING:**

This descriptive cross-sectional study was conducted at the Faculty of Health Sciences of a university in Turkey.

**METHODS:**

The data of this study were collected from digital media between July and October 2020 from a study population of 385 students. The data were collected using the Personal Information Form, including the socio-demographic characteristics of students, the Sexual and Reproductive Health Stigmatization Scale in Young Women and the Perception of Gender Scale. Descriptive statistics, independent samples t-test, analysis of variance, and Pearson’s correlation test were used to assess the data.

**RESULTS:**

It was determined that there was a negative correlation between the Sexual and Reproductive Health Stigmatization Scale in Young Women and the Perception of Gender Scale (r = -0.173, P = 0.001).

**CONCLUSION:**

It was determined that as the gender perception in the young women who participated in the study increased, the sexual and reproductive health stigmatization level decreased. The sexual and reproductive health stigmatization levels of the participants were at an above average level, and gender perception was at a medium level.

## INTRODUCTION

Social norms determine the sexual behavior, marriage traditions, punishments for unapproved sexual behavior, prevention of pregnancy, sex education, homosexuality, and attitudes concerning sexual taboos.^1^ According to these norms, the sexual behavior of young individuals and pregnancy, abortion, premature birth, and sexually transmitted infections, which occur as a result of such behavior, are defined as immoral according to the social, cultural, and religious norms in some societies and cause the individual to be stigmatized.^2,3^

Stigmatization is a condition preventing young women from using birth control methods and services.^4^ Stigmatization that emerges depending on the sexual behavior of young individuals leads to negative health and social outcomes, such as shame, social marginalization, violence, and mental health morbidity worldwide.^5^ The inability of young people to benefit from reproductive health and counseling services due to stigmatization increases unsafe miscarriages and maternal mortality.^6^

Gender role, which is defined as the individual’s perception of himself/herself as a woman or a man and the exhibition of behaviors required by his/her sex, is taught to the individual according to the moral principles of his/her society. He/she is expected to behave in line with this role.^1^ The role of protecting the “family’s honor,” which is attributed to women, causes women to be accused in all kinds of sexual relations. In addition, a reason for honor killings is when single women experience their sexuality against the roles that are expected from them. Moreover, pre-marital sexual intercourse experienced by single women in Turkey is one of the reasons for honor killings.^7^

Under the influence of gender inequality, sexuality in societies such as that of Turkey is associated with marriage for women, whereas for men it becomes an expected and acclaimed activity.^7^ While women are completely forbidden to have sexual intercourse before marriage, men in the same societies are encouraged to have it.^8^ Therefore, women, who experience their sexual life within the boundaries allowed by society, are under the inspection of society.^9^ Morever, the pressure created by society leads to hymen control, adolescent or unintended pregnancies, miscarriages under unhealthy conditions, and an inability of benefiting from healthcare services.^7^ Virginity control, which is another application containing gender discrimination, is observed in most traditional societies. Virginity examination, which has been created as a means of controlling the sexuality of women, leads to mental and physical problems in women, besides honor killings and suicides because it removes the voice of women over their own body.^8^

The knowledge and behaviors of young people going to university in Turkey concerning sexuality are different from married young people. A significant part of university students is single, and most are men and minors; however, a significant part of women experience the first contact and sexual intercourse with the opposite sex.^10^ In the literature, we have not encountered studies examining the correlation between sexual and reproductive health stigmatization and perception of gender in young women.

## OBJECTIVE

Therefore, in this study, we aimed to examine the correlation between the sexual and reproductive health stigmatization level and perception of gender in female university students. In line with this purpose, we believe that the present study would contribute to the literature.

### Research questions

- Is there a difference between the socio-demographic characteristics of the participants and their sexual and reproductive health stigma levels?
- Is there a difference between the socio-demographic characteristics of the participants and their gender perceptions?
- Is there a relationship between the stigma of sexual and reproductive health and perception of gender?

## METHODS

The population of the descriptive cross-sectional study comprised 615 female students receiving education in the Faculty of Health Sciences of a university in Turkey. The simple random sampling method, one of the non-probability sampling methods, was used in the study. While calculating the sample size of the study, the sampling method with a known universe was used. The sample of the study is; it was calculated as 237 individuals according to 5% margin of error and 95% confidence interval. However, since it was planned to reach the entire universe, the study was conducted with a total of 385 female students who volunteered to participate. The data were collected via a web-based survey form in digital media between July and October 2020. The digital survey form was shared with students in social media platforms, such as Whatsapp, Instagram, and Twitter. The online form allowed students to give only one answer. It took nearly 10 minutes to complete the survey form.

### Inclusion criteria

- Being of the female sex and aged between 18 to 24 years,
- Receiving education in the Faculty of Health Sciences in the university where the study was conducted, and
- Being able to use social networks.

### Exclusion criteria

- Individuals who were not students in the Faculty of Health Sciences of the university where the research was conducted, who were not women between the ages of 18–24 years, and who could not use social networks were not included in the study.

### Data collection form

The “Personal Information Form,” the “Sexual and Reproductive Health Stigmatization Scale in Young Women,” and the “Perception of Gender Scale” were used as the data collection forms.

### Personal information form

Prepared by the researchers in line with the literature,^1,7,10^ this form comprised a total of 13 questions containing the socio-demographic characteristics of students, such as age, grade, parental education, number of siblings, and the region they lived in. There were no open-ended questions in the “Personal Information Form”.

### Sexual and Reproductive Health Stigmatization Scale in Young Women (SRHSSYW)

A scale developed by Hall et al.^5^ in 2017 to determine the stigmatization associated with the sexual and reproductive health in women aged 15 to 24 years. The Turkish validity study of the scale was conducted by Bayrakçeken in 2018.^11^ The original version of the scale has three subscales and 20 items. The sub-scales of the scale are; “Accepted Stigmatization,” “Internalized Stigmatization,” and “Stigma-based Attitudes.” The three-point likert scale is rated as 0 = disagree, 0 = neutral, and 1 = agree. The lowest and highest scores to be obtained from the overall scale are 0 and 20, respectively. Higher scores indicate an increase of stigmatization. The Cronbach’s alpha value of the scale is 0.74.^11^ In this study, the Cronbach’s alpha reliability coefficient of the scale was found to be 0.758.

### Perception of Gender Scale (PGS)

A five-point likert scale [strongly agree (5), agree (4), undecided (3), disagree (2), and strongly disagree (1)] with 25 items was developed to evaluate gender perception of individuals. Of the items, 10 were written positively, whereas 15 were written negatively.^12^ The lowest and highest scores to be obtained from the scale were 25 and 125, respectively. Higher scores indicated a “positive” gender perception, while lower scores indicated a “negative” gender perception. The Cronbach’s alpha reliability coefficient of the scale was 0.872.^13^ In this study, the Cronbach’s alpha reliability coefficient of the scale was found to be 0.792.

### Statistical analysis

The data were evaluated using the IBM SPSS statistics software, version 22 (IBM SPSS, Osmaniye, Turkey). First, the convenience of the data for normal distribution was evaluated using a Skewness and Kurtosis (±1) distribution test. All of the data were found to be normally distributed. An independent samples t-test and analysis of variance test, alongside descriptive statistics (percentage, frequency, mean, standard deviation, minimum, and maximum) were applied to assess the data. A Pearson correlation analysis was used to measure the relationship between the Stigma of Young Women’s Sexual and Reproductive Health Stigma Scale and Gender Perception Scale total and sub-dimension scores.

### Ethical considerations

To conduct the study, ethics committee approval (Date of approval: June 22, 2020/ Ethics committee no: E.454), institutional permission, and permissions for use of the scales were received. There was information about the purpose and content of the study and voluntary basis of the study in the survey, which was submitted to the participants. The survey did not record the the identity-related information of the participants. This study was conducted in accordance with the Declaration of the Principles of Helsinki.

## RESULTS

Of the students who participated in the study, 87% were aged 19 years and above, 37.9% were the 2nd-year students, mothers of 80% of the students were primary school graduates, mothers of 89.9% were housewives, fathers of 56.4% were primary school graduates, fathers of 32.5% were self-employed, and 53% of the students had 4 or more siblings. It was determined that 77.7% of the students had an income equal to the expense, 81.3% had a monthly allowance under 1000 Turkish lira, 47.8% lived in the Mediterranean Region, 56.4% lived in a province, and 88.6% lived with their parents (Table 1).

**Table 1.** Distribution of the socio-demographic characteristics of the individuals (n = 385)

		n	%
**Age**	Under 19 years	50	13
	19 years and above	335	87
**Year**	1st year	106	27.5
	2nd year	146	37.9
	3rd year	74	19.2
	4th year	59	15.3
**Mother’s** **education**	≤ Primary education	308	80
	High school	58	13.1
	≥ University	19	4.9
**Mother’s** **profession**	Housewife	346	89.9
	Civil servant	10	2.6
	Worker	23	6
	Self-employed	6	1.6
**Father’s** **education**	≤ Primary education	217	56.4
	High school	111	28.8
	≥ University	57	14.8
**Father’s** **profession**	Civil servant	111	28.8
	Worker	114	29.6
	Self-employed	125	32.5
	Unemployed	35	9.1
**Number of** **siblings**	Not applicable	8	2.1
	1 sibling	87	22.6
	2-3 siblings	86	22.3
	≥ 4 siblings	204	53.0
**Income** **status**	Income less than expense	49	12.7
	Income equal to expense	299	77.7
	Income more than expense	37	9.6
**Monthly** **allowance**	Under 1000 ₺	313	81.3
	1000-2000 ₺	52	13.5
	Above 2000 ₺	20	5.2
**Region lived**	Mediterranean Region	184	47.8
	Aegean Region	30	7.8
	Central Anatolia Region	76	19.7
	Black Sea Region	9	2.3
	Eastern/Southeastern Anatolia Region	86	22.3
**Place of** **residence**	Province	217	56.4
	District	110	28.6
	Rural	58	15
**People they** **lived with**	Parents	341	88.6
	Mother	19	4.9
	Father	8	2.1
	Family elders	17	4.4
	**Total**	**385**	**100.0**

₺ = Turkish lira.

It was determined that the difference between the PGS total and SRHSSYW total and subscale mean scores of the students who took part in the study was not statistically significant according to their age, mother’s profession, father’s education, income status, region lived, and place of residence (P > 0.05). There was a statistically significant difference between the Accepted Stigmatization subscale scores of the participants according to their grade; between the Stigma-based Attitudes subscale scores according to their mother’s education; between the PGS total mean scores according to their father’s profession and number of siblings; and between the SRHSSYW total mean scores according to their monthly allowance (P < 0.05) (Table 2).

**Table 2.** Distribution of the socio-demographic characteristics and the Sexual and Reproductive Health Stigmatization Scale in Young Women (SRHSSYW) and Perception of Gender Scale (PGS) total and subscale mean scores of the individuals

		Accepted stigmatization	Internalized stigmatization	Stigma-based attitudes	Total SRHSSYW	PGS
		$\bar{\text{X}}\;\pm$	$\bar{\text{X}}\;\pm \;$	$\bar{\text{X}}\;\pm \;$	$\bar{\text{X}}\;\pm \;$	$\bar{\text{X}}\;\pm \;$
**Age**	≤ 19 years	4.58 ± 1.55	3.54 ± 1.4	6.18 ± 2.33	14.3 ± 4.02	66.76 ± 4.81
	≥ 19 years	4.73 ± 1.36	3.67 ± 1.3	6.08 ± 2.37	14.4 ± 3.67	66.85 ± 4.88
	**Significance** ^a^	P = 0.456	P = 0.511	P = 0.788	P = 0.733	P = 0.902
**Year**	1st year	4.64 ± 1.40	3.86 ± 1.25	5.85 ± 2.29	14.36 ± 3.64	67.12 ± 5.17
	2nd year	4.73 ± 1.40	3.67 ± 1.29	6.21 ± 2.36	14.61 ± 3.65	66.85 ± 5.08
	3rd year	4.83 ± 1.27	3.29 ± 1.46	5.97 ± 2.38	14.10 ± 3.75	65.71 ± 4.11
	4th year	4.66 ± 1.48	3.67 ± 1.23	6.38 ± 2.45	14.72 ± 3.99	67.69 ± 4.45
	**Significance** ^b^	P = 0.805	**P = 0.041**	P = 0.471	P = 0.732	P = 0.105
**Mother’s education**	≤ Primary education	4.69 ± 1.39	3.63 ± 1.30	5.94 ± 2.36	14.27 ± 3.37	67.02 ± 4.59
	High school	4.89 ± 1.29	3.79 ± 1.28	6.62 ± 2.36	15.31 ± 3.45	65.58 ± 5.93
	≥ University	4.47 ± 1.57	3.52 ± 1.64	7.0 ± 1.94	15.0 ± 3.29	67.63 ± 5.16
	**Significance** ^b^	P = 0.448	P = 0.448	**P = 0.031**	P = 0.123	P = 0.090
**Mother’s profession**	Housewife	4.71 ± 1.40	3.63 ± 1.31	6.02 ± 2.38	14.36 ± 3.76	66.90 ± 4.85
	Civil servant	4.80 ± 0.91	4.00 ± 1.33	6.30 ± 2.31	15.10 ± 3.87	66.20 ± 4.84
	Worker	5.00 ± 1.16	4.00 ± 1.41	6.78 ± 2.19	15.78 ± 2.95	66.39 ± 5.50
	Self-employed	3.83 ± 1.60	3.00 ± 1.41	7.16 ± 1.47	14.0 ± 2.82	65.83 ± 3.65
	**Significance** ^b^	P = 0.331	P = 0.281	P = 0.316	P = 0.322	P = 0.877
**Father’s education**	≤ Primary education	4.76 ± 1.33	3.64 ± 1.27	5.98 ± 2.35	14.39 ± 3.19	67.0 ± 4.95
	High school	4.56 ± 1.49	3.61 ± 1.42	6.27 ± 2.17	14.45 ± 3.59	66.5 ± 4.42
	≥ University	4.82 ± 1.37	3.78 ± 1.30	6.15 ± 2.73	14.77 ± 3.97	66.8 ± 5.36
	**Significance** ^b^	P = 0.390	P = 0.695	P = 0.557	P = 0.790	P = 0.679
**Father’s profession**	Civil servant	4.82 ± 1.24	3.68 ± 1.29	6.24 ± 2.51	14.75 ± 3.61	65.72 ± 4.51
	Worker	4.63 ± 1.39	3.57 ± 1.40	6.00 ± 2.43	14.21 ± 3.81	66.94 ± 4.89
	Self-employed	4.72 ± 1.45	3.64 ± 1.31	5.94 ± 2.26	14.31 ± 3.80	67.43 ± 4.78
	Unemployed	4.62 ± 1.59	3.82 ± 1.17	6.48 ± 1.96	14.94 ± 3.44	67.88 ± 5.65
	**Significance** ^b^	P = 0.732	P = 0.792	P = 0.557	P = 0.573	**P = 0.025**
**Number of siblings**	N/A	4.87 ± 1.35	3.12 ± 1.64	7.12 ± 1.45	15.12 ± 4.05	67.12 ± 4.79
	1 sibling	4.67 ± 1.36	3.74 ± 1.17	6.49 ± 2.29	14.91 ± 3.41	65.31 ± 5.47
	2-3 siblings	4.95 ± 1.21	3.76 ± 1.36	6.06 ± 2.56	14.79 ± 3.77	67.37 ± 5.07
	≥ 4 siblings	4.62 ± 1.46	3.58 ± 1.34	5.89 ± 2.31	14.11 ± 3.79	67.25 ± 4.38
	**Significance** ^b^	P = 0.320	P = 0.415	P = 0.142	P = 0.257	**P = 0.011**
**Income status**	Income less than expense	5.08 ± 1.11	3.64 ± 1.18	6.05 ± 2.43	14.78 ± 3.51	66.27 ± 4.35
	Income equal to expense	4.70 ± 1.37	3.60 ± 1.38	6.08 ± 2.39	14.39 ± 3.76	66.89 ± 5.04
	Income more than expense	4.51 ± 1.63	3.97 ± 0.96	6.20 ± 2.17	14.69 ± 3.62	66.93 ± 4.13
	**Significance** ^b^	P = 0.161	P = 0.179	P = 0.941	P = 0.751	P = 0.756
**Monthly allowance**	Under 1000 ₺	4.65 ± 1.39	3.66 ± 1.33	6.05 ± 2.34	14.38 ± 3.71	67.01 ± 4.56
	1000-2000 ₺	5.09 ± 1.08	3.76 ± 1.13	6.61 ± 2.26	115.48 ± 3.30	65.98 ± 6.40
	Above 2000 ₺	4.65 ± 1.84	3.20 ± 1.43	5.35 ± 2.70	13.20 ± 4.40	66.30 ± 4.85
	**Significance** ^b^	P = 0.109.	P = 0.249	P = 0.101	**P = 0.041**	P = 0.321
**Region lived**	Mediterranean Region	4.65 ± 1.44	3.67 ± 1.23	6.26 ± 2.16	14.59 ± 3.56	66.70 ± 4.95
	Aegean Region	4.90 ± 1.24	3.60 ± 1.27	6.36 ± 2.23	14.86 ± 2.86	64.66 ± 3.67
	Central Anatolia Region	4.73 ± 1.34	3.69 ± 1.36	5.77 ± 2.69	14.21 ± 3.92	67.27 ± 4.73
	Black Sea Region	5.88 ± 0.33	3.88 ± 1.16	7.77 ± 1.09	17.55 ± 1.66	66.44 ± 5.61
	Eastern/Southeastern Anatolia Region	4.65 ± 1.38	3.55 ± 1.49	5.75 ± 2.51	13.96 ± 4.13	67.53 ± 4.94
	**Significance** ^b^	P = 0.109	P = 0.920	P = 0.060	P = 0.068	P = 0.073
**Place of residence**	Province	4.67 ± 1.31	3.58 ± 1.32	6.11 ± 2.29	14.38 ± 3.61	66.53 ± 4.83
	District	4.85 ± 1.43	3.73 ± 1.33	6.10 ± 2.52	14.70 ± 3.84	67.02 ± 4.86
	Rural	4.60 ± 1.56	3.74 ± 1.27	5.98 ± 2.35	14.32 ± 3.88	67.63 ± 4.95
	**Significance** ^b^	P = 0.441	P = 0.551	p = 0.924	P = 0.737	P = 0.273
**Home residents**	Parents	4.74 ± 1.33	3.64 ± 1.31	6.08 ± 2.34	14.47 ± 3.67	66.86 ± 4.88
	Mother	4.42 ± 1.62	4.00 ± 1.10	7.00 ± 1.59	15.42 ± 2.93	66.36 ± 3.78
	Father	4.50 ± 2.07	4.12 ± 1.12	7.12 ± 1.55	15.75 ± 4.13	65.75 ± 3.15
	Family elders	4.64 ± 1.76	3.17 ± 1.59	4.76 ± 3.23	12.58 ± 4.70	67.35 ± 6.37
	**Significance** ^b^	P = 0.752	P = 0.208	**P = 0.021**	P = 0.087	P = 0.856

SD = Standard deviation, P < 0.05 (^a^independent samples t-test, ^b^analysis of variance); ₺ = Turkish lira.

Bold values indicate statistical signifcance.

It was determined that the SRHSSYW total mean score was 14.46 ± 3.71 and the Accepted Stigmatization subscale mean score was 4.71 ± 1.38; the Internalized Stigmatization subscale mean score was 3.65 ± 1.31; additionally, the Stigma-based Attitudes subscale mean score was 6.09 ± 2.36 and the PGS mean score was 66.83 ± 4.86 (Table 3).

**Table 3.** Distribution of the PGS and SRHSSYW total and subscale mean scores and minimum-maximum values

	$\bar{\text{X}}$	SD	Min-Max received
**Total SRHSSYW**	14.46	3.71	0-20
Accepted Stigmatization	4.71	1.38	0-6
Internalized Stigmatization	3.65	1.31	0-5
Stigma-based Attitudes	6.09	2.36	0-9
**Total PGS**	66.83	4.86	25-125

PGS = Perception of Gender Scale; SRHSSYW = Sexual and Reproductive Health Stigmatization Scale in Young Women; SD = standard deviation; Min-Max = minimum-maximum.

It was determined that there was a negative weak correlation between the SRHSSYW and the PGS (r = -0.173, P = 0.001). In other words, as the gender perception in young women increases, the sexual and reproductive health stigmatization level decreases. It was determined that there was a negative correlation between the subscales of the SRHSSYW and the PGS (Table 4).

**Table 4.** Correlation distribution of the Perception of Gender Scale (PGS) scores and Sexual and Reproductive Health Stigmatization Scale in Young Women (SRHSSYW) total and subscale scores (n = 385)

		1	2	3	4
1 SRHSSYW Total		r P				
2 Accepted Stigma		r P	0.620^**^ **0.001**			
3 Internal Stigma		r P	0.848^**^ **0.001**	0.261^**^ **0.001**		
4 Stigma-based Attitudes		r P	0.646^**^ **0.001**	0.228^**^ **0.001**	0.324^**^ **0.001**	
5 PGS Total		r P	−0.173^**^ **0.001**	−0.135^**^ **0.008**	−0.181^**^ **0.001**	−0.021 0.678

P < 0.01 (^**^Correlation test); ^**^Correlation is significant at the level of 0.01 (2-tailed).

Bold values indicate statistical signifcance.

## DISCUSSION

Stigmatization is defined as a key social determinant of health and a driving force of health inequalities.^14^ Stigmatization is conceptualized as a quality which turns humans from whole and ordinary individuals into imperfect and despised individuals and disgraces them.^4^ In sexual health and reproductive health (SHRH), social, cultural, and religious norms, which enframe the sexual behaviors of adolescents and their consequences (pregnancy, early childbirth, abortion, and sexually transmitted infections) to be immoral and problematical, lead to stigmatization.^2,15^

In this study, it was seen that factors, such as age, mother’s profession, father’s education, income status, and region lived did not affect the gender perception and reproductive health stigmatization perception level in young women. Another study suggested that factors, such as age, city, religious belief, educational background, relationship status, unemployment, health and sexual relationship histories graded by oneself, receiving family planning services, use of modern contraceptives, number of pregnancies, and sexual intercourse, affected the SHRH stigmatization perception.^5^ Additionally, in studies, it is stressed that age, marital status, income, place of residence of individuals, and socio-demographic and cultural factors especially when it comes to those who are unmarried, pose a great obstacle to benefiting from reproductive health services due to the fear of stigmatization.^16,17^ A previous study conducted in Iran demonstrated that the fear of stigmatization was the greatest obstacle to benefiting from reproductive health services.^18^ The basis of stigmatization is formed by prejudices and beliefs. The social, cultural, and religious norms define sexual behaviors of adolescents and their consequences (such as pregnancy, early childbirth, abortion, and sexually transmitted infections) to be immoral, and this causes the individual to be stigmatized.^2^

In the study, the sexual health stigmatization perception of the young people was higher according to the mother’s education, number of siblings, and monthly allowance. It was observed that the socioeconomic level of the family affected this perception. No matter from what standpoint it is viewed, physical and psychosocial welfare has a profoundly negative impact on reproductive health stigmatization perception.^19,20^ This may restrain the health and development of young people.^21^ The occurrence of stigmatization is closely associated with the context and structure of society.^22^ Stigmatization is experienced when an individual or a group is defined differently from a perceived norm and is subjected to labeling, shame, disapproval, and discrimination.^14^ When social circumstances restrain the welfare and access to opportunities and resources, the access to healthcare services and quality care is also restrained, while the social, cultural, and gender norms hardly affect stigmatization. Notably, community-based or belief-based organizations or politicians play a key role in sustaining or struggling with stigmatization.^23^

Upon examining the literature, it has been shown that stigma-tization, which is attributed to unmarried women benefiting from reproductive health services, contains situations, such as stereotypes, fear of being labeled, discrimination, and shame of receiving reproductive health services. In South Asian countries, where premarital sexual relationships are forbidden^24^ and a woman’s pre-marital virginity status is valued very much, the procurement of reproductive health services to unmarried women causes significant exposure from these cultures.^25^ The belief that a reproductive health problem experienced by a young woman may be associated with sexual relationships causes the woman to be stigmatized and rejected by the society.^26^ Stereotypical thoughts and assumptions concerning this issue lead to stigmatization and make it difficult for unmarried individuals to access reproductive health services.

There is an increasing interest in promoting gender equality and Women’s and Girls’ Empowerment as a way of accelerating the progress and enhancing women’s health and welfare by accepting the restrictions of unequal gender power dynamics in a woman’s life.^27^ According to the results of the study, negative gender perception had a negative effect on sexual and reproductive health stigmatization level. Examining similar studies, it is indicated that the society associates reproductive health matters with sexual relationships, and this shapes stigmatization.^28^ In their study, Rehnström Loi et al. indicated that more than 50% of Kenyan secondary school students have stigmatizing attitudes toward abortion and the use of contraceptive methods. Students of age 13–15 years and male students have a higher potential of having stigmatizing views.^29^ Another study conducted in Nepal stated that cultural and gender norms were factors increasing stigmatization and discrimination.^30^ This can be associated with the fact that the views of young people are influenced by social norms and cultural traditions.

The studies have stated that the SHRH understanding and perception of young women coincide with a variety of stigmatization areas. These areas are sex, pregnancy, childbirth and abortion, stigmatization of adolescent girls as “immoral,” “disrespectful,” and “disobedient” by society, description of adolescent girls as “mean girls” by community, stigmatization or gossip applied to young women, and negative attitudes arising from marginalization and maltreatment are shame and guilt felt by young women as a result of legalized stigmatization. Due to the stigmatization, these situations also prevent young women from using contraceptive methods and services.^15^

### Limitations

This study was limited to female students, who were of age between 18 and 24 years and were enrolled at the Faculty of Health Sciences of a Turkish university, used social networks, and agreed to participate in the study. The other limitations of our research are the facts that the data collection process coincided with the pandemic period and the study was carried out within a certain time period.

## CONCLUSION

According to the results of this study, factors, such as age, mother’s profession, father’s education, income status, region lived, and place of residence, do not affect the sexual and reproductive health stigmatization and gender perception of young women. However, socioeconomic factors, such as the mother’s level of education, father’s profession, number of siblings, and monthly allowance affect the stigmatization perception. The increase of gender perception in young women decreases the sexual and reproductive health stigmatization level.

In line with the results, it is important that women and girls be empowered for SHRH and global development goals, especially concerning the increasing gender equality. It is also important that universal access to SHRH services be included within the scope of healthcare services. Society, families, and unmarried women themselves should understand that sexual and reproductive health is an important part of the whole life cycle of a woman. It is necessary to overcome the prejudice that conditions related to reproductive health are certainly associated with the conclusion that a person is having sexual intercourse, to provide reproductive health service as part of health in every period of life and provide an equality of opportunity to young women to benefit from this service. It is recommended to carry out similar studies in different regions of Turkey, to plan training programs in line with the results obtained, and to establish research programs to combat the stigma.
